# Supplementary material for: The effect of gamma irradiation on the stability of vitamin D in select finfish species
Source: PLoS One. 2025 Sep 26;20(9):e0332867. doi: 10.1371/journal.pone.0332867 (PMC12469152; doi:10.1371/journal.pone.0332867)
Supplement: Attachment — Submitted filename: review-TL.docx [file pone.0332867.s001.docx]

1. The focus of this manuscript is on the study of the effect of irradiation on vitamin D in fish, but there is literature reporting that irradiation can affect the structure of the substance. Please add characterization of the structure of vitamin D and assess whether there is an effect of different irradiation doses on the structure of vitamin D and thus the function of the vitamin D.

2. Since irradiation has a certain penetration distance for sterilization, please add a flow chart of the process of actually irradiating a fish product. Because the size of the irradiation dose varies for different thicknesses of irradiated material, the structure of vitamin D may be destroyed in order to obtain a sterilizing dose.

3. Line32-42 The focus of this manuscript should be on the importance of vitamin D and the significance of using gamma radiation. If the effects of Vibrio on fish are to be illustrated, the number of Vibrio should be measured after the method is used.

4. Line43-50 List the current methods of preservation used in the transportation of fish and their advantages and disadvantages, followed by a description of the advantages of the methods used in this manuscript.

5. Line49-50 Use of the method for other articles is accompanied by corresponding literature.

6. Line126-137 Whether the effect of different gamma rays on pure vitamin D should be set.

7. Line200-201 Provide relevant literature that does not alter sensory properties.

8. Line208-209 Appropriate sensory tests including color, odor, etc. should be provided.

9. Line213-227 Will the treatment cause adverse effects and within what range will it not cause adverse effects with references.
