# Supplementary material for: The effect of gamma irradiation on the stability of vitamin D in select finfish species
Source: PLoS One. 2025 Sep 26;20(9):e0332867. doi: 10.1371/journal.pone.0332867 (PMC12469152; doi:10.1371/journal.pone.0332867)
Supplement: Attachment — Submitted filename: finfish_reponse_to_reviewers.docx [file pone.0332867.s002.docx]

Dear Dr. Mitra,

While we would like to thank you and the reviewer for evaluating our manuscript, we believe that you and the reviewer have made fundamental errors in your assessment, not abided by the review standards set forth by PLoS One, and have simply asked us ‘to do more work’ that is clearly outside the scope of our manuscript.

PLoS One evaluates based on technical rigor and not novelty. As listed on their website, the criteria for review are the following:

*1. The study presents the results of original research.* **Our work and data are entirely novel as they detail the effects of irradiation on vitamin D in commercially important finfish species.**

2. *Results reported have not been published elsewhere.* **Our results have not been published elsewhere.** They are entirely novel.

3. *Experiments, statistics, and other analyses are performed to a high technical standard and are described in sufficient detail.* **Not a single comment below indicates that the technical aspects of the proposal are flawed.** We have applied appropriate statistics, including correcting for multiple comparisons, have sufficiently high replication, our experiments and analyses were performed according to industry standards.

4. *Conclusions are presented in an appropriate fashion and are supported by the data.* Our conclusion, as indicated in the discussion and abstract, is that irradiation can affect the stability of vitamin D in a dose-dependent, temperature-dependent, and fish species-specific data. This conclusion is well supported by the data presented in Figures 2 and 3, and is supported by a statistical analysis. Further, **not a single comment below challenged the fundamental conclusions made in our manuscript.**

5. *The article is presented in an intelligible fashion and is written in standard English*. Our manuscript was written and edited by authors with over 50 publications. **Not a single comment below indicates that our manuscript needs to be better written;** it only indicates that more work needs to be done for, quite frankly, the sake of asking for more work as it is clearly outside of the scope of the manuscript.

6. *The research meets all applicable standards for the ethics of experimentation and research integrity*. Not a single comment below indicated any issues with ethics. In fact, we supplied all of our raw data for the reviewers to analyze, including calibration curves (Fig. 1). **This far exceeds the standard of the community.**

7. *The article adheres to appropriate reporting guidelines and community standards for data availability.* **Not a single comment below indicates that our data is not reported correctly or does not meet the community standard of availability**. All of our raw data is indeed included in the manuscript.

As further evidence that both you and reviewer did not follow the standard of review and assessment as set forth by PLoS One, **the reviewer indicated that all of the above was fine in their evaluation.** Directly from the reviewer report:

**Comments to the Author**

1. Is the manuscript technically sound, and do the data support the conclusions?

Reviewer #1: Yes

2. Has the statistical analysis been performed appropriately and rigorously?

Reviewer #1: Yes

3. Have the authors made all data underlying the findings in their manuscript fully available?

Reviewer #1: Yes

4. Is the manuscript presented in an intelligible fashion and written in standard English?

Reviewer #1: Yes

**Thus, all of the criteria required for publication in *PLoS One* have been met.** What remains are comments from the reviewer that lack a scientific basis and clarity, are outside the scope of our manuscript, or are so minor in their scope that 10 minutes in a document editing system could fix the issue. **Certainly, none of these comments, either alone or as a synthesis, warrants a rejection as we have clearly met the review criteria for *PLoS One* as indicated by the reviewer themselves in the report above.**

Our responses to the reviewer comments are below.

1. The focus of this manuscript is on the study of the effect of irradiation on vitamin D in fish, but there is literature reporting that irradiation can affect the structure of the substance. Please add characterization of the structure of vitamin D and assess whether there is an effect of different irradiation doses on the structure of vitamin D and thus the function of the vitamin D.

**This is outside of the scope of our manuscript.** The central focus of our manuscript is the stability of vitamin in finfish after irradiation. As clearly indicated in the introduction:

“While irradiation represents an established strategy that could reduce the incidence of

pathogens found in finfish, it remains unclear as to how irradiation impacts the stability of vitamin D in finfish tissue of commercial species.”

This is exactly what we have studied and precisely what we have reported on.

Our central conclusion, as clearly indicated in the abstract is:

“Overall, our results indicate that irradiation of finfish may not reduce vitamin D concentration when applied at dosages of 0.5 kGy and 2 kGy or less in chilled and frozen conditions, respectively.”

And, from our discussion:

“In conclusion, we found that irradiating select finfish species could reduce the vitamin D concentration in a species, dose, and temperature-dependent manner. Together, these findings suggest that irradiation at appropriate intensities can reduce the pathogenic burden on finfish while maintaining the concentration of vitamin D.”

**We make no claim on the structure of vitamin D, nor does it matter to any single conclusion made in our manuscript**. This comment does not impact the technical rigor or conclusions made in the manuscript and is thus entirely unwarranted and outside the scope of our manuscript.

The reviewer is correct that the effects of ionizing radiation on vitamin D have been previously studied. In fact, it is commonly known that to produce vitamin D, ionizing radiation is required. The steps in this process have been well documented ^1^. Accordingly, there is no reason to reproduce this data in this manuscript.

^1^

Finally, Vitamin D was accurately identified by comparing its retention time to a pure chemical standard and confirming the match through molecular and fragment ion patterns in the mass spectral data. This community standard for measuring vitamin D has been used multiple times before ^2-12^. We have applied the most rigorous methods to detect vitamin D.

**Taken together, this comment is a) outside the scope of the manuscript, b) does not challenge the fundamental analysis or conclusions of the manuscript, and c) provides no value to the manuscript if performed.**

2. Since irradiation has a certain penetration distance for sterilization, please add a flow chart of the process of actually irradiating a fish product. Because the size of the irradiation dose varies for different thicknesses of irradiated material, the structure of vitamin D may be destroyed in order to obtain a sterilizing dose.

We have included both the dosimetry data and a figure showing the irradiation process (Figure 1). This new material can be found in the methods section.

However, the second sentence of the reviewer’s comment is entirely unclear. We have no interest and make no claim on sterilizing doses. That is not a conclusion of our manuscript, nor is it a goal. Determining a sterilizing will have not change the fundamental conclusions of our manuscript, nor will it add anything to the manuscript within its scope.

Further to that, many, if not all, papers that have looked at the stability of vitamins and nutrients in finfish or other matrices do not identify sterilizing dose ^13-30^. Why? **Because it is entirely unnecessary and outside of the scope of this and the work of others.**

**We also note that sterilizing doses for multiple finfish species have been established elsewhere ^31^. Thus, this work is superfluous to what has already been produced by the community.**

In summary, we added a flow chart and dosimetry data. However, **determining a sterilizing dose is completely unnecessary within the scope of the manuscript and will have no fundamental impact on our conclusion or data analysis.**

3. Line32-42 The focus of this manuscript should be on the importance of vitamin D and the significance of using gamma radiation. If the effects of Vibrio on fish are to be illustrated, the number of Vibrio should be measured after the method is used.

We included this information in our original manuscript to highlight the use of gamma irradiation on fish due to concerns about pathogenic bacteria, *Vibrio* being chief amongst them. Since numerous studies have already demonstrated that gamma irradiation effectively eliminates *Vibrio* in fish, we did not repeat these experiments. In light of the reviewer’s comment, we have removed this paragraph.

4. Line 43-50 List the current methods of preservation used in the transportation of fish and their advantages and disadvantages, followed by a description of the advantages of the methods used in this manuscript.

Our primary focus is on vitamin D stability and irradiation of finfish, not on all preservation and transportation methods of finfish; that material belongs in a review article. Adding this information will only muddy the waters in terms of the central focus of our manuscript, will not fundamentally enhance readability, or will not change our analysis or conclusions. In addition, many manuscripts have been published on the irradiation of foods, and the vast majority do not summarize this information as it is superfluous and obscures the central message of these publications ^2-12^.

5. Line 49-50 Use of the method for other articles is accompanied by corresponding literature.

**It is not clear what the reviewer is asking for here.** We think that they might be asking for references indicating approval for the irradiation of foods in the United States. We have included these references.

6. Line 126-137 Whether the effect of different gamma rays on pure vitamin D should be set.

This has already been determined previously, **as noted in our original manuscript**.

*“Previous work performed on whole extracts of finfish^32^,* ***vitamin D resuspended in organic solvents^32^****, and chicken^25^ has yielded somewhat conflicting results. On the one hand, some studies show that irradiation does not impact the concentration of vitamin D ^33^. On the other, some previous work has shown that irradiation can reduce the concentration of vitamin D ^5,32^”*

For example, previous work has assessed the stability of pure vitamin D in benzene, ethanol, cod oil, liver oil, and butter ^5,32^. This synthesis of work has clearly established the effects of irradiation on pure vitamin D. Our work establishes the impact of irradiation on vitamin D in finfish and is entirely different from this previous work.

Therefore, why repeat this work? It would not fundamentally alter our manuscript's technical rigor, conclusions, or anything else. Once again, this is a reviewer comment that asks for more work for the sake of asking for more work. **It is entirely irrelevant to our manuscript.**

Moreover, nearly all other manuscripts investigating the stability of a vitamin or nutrient in foods do not look at the purified product ^13-27^. Why? **Because, as clearly stated in our introduction, the matrix of the food can determine the stability** ^34^.

***“****However, to date, not a single study has investigated the stability of vitamin D in fresh, commercially important finfish species. This is important to consider as the composition of  the chemical matrix where vitamin D is found may react differently to irradiation and may provide protection against any loss.”*

The premise of the above statement has been established in multiple studies and in multiple foods (reviewed in ^34^), but not commercially important finfish, which we are examining here as demonstrated by our specific questions outlined in the introduction

*“While irradiation represents an established strategy that could reduce the incidence of pathogens found in finfish, it remains unclear as to how irradiation impacts the stability of vitamin D in finfish tissue of commercial species.”*

**Thus, not only is the proposed work entirely outside of the scope of the manuscript, but it is entirely contradictory to the specific question we asked, as indicated in the introduction.**

**To clarify that this work has already been completed, the introduction now reads:**

*“Previous work performed on whole extracts of finfish^32^,* ***purified vitamin D resuspended in organic solvents^32^****, and chicken^25^ has yielded somewhat conflicting results. On the one hand, some studies show that irradiation does not impact the concentration of vitamin D ^33^. On the other, some previous work has shown that irradiation can reduce the concentration of vitamin D ^5,32^”*

7. Line 200-201 Provide relevant literature that does not alter sensory properties.

**It is not clear what the reviewer is requesting here**. However, we think that they are asking to provide a reference about the effects of irradiation on organoleptic properties. While this was discussed in depth and with references in the paragraph, we have removed the paragraph in response to the previous comment.

8. Line 208-209 Appropriate sensory tests including color, odor, etc. should be provided.

**This is entirely outside the scope of our manuscript.** **This suggested work will have absolutely no impact on our technical rigor or fundamental conclusions.** Moreover, multiple other studies that have examined the effect of irradiation on nutrients and vitamins do not do this ^14,17,18,25-30^.

*The reviewer is simply asking for more work for the sake of asking for more work.* We make absolutely no claim on the organoleptic properties of finfish; simply that we noticed changes and that they need to be evaluated elsewhere and carefully chosen. As indicated in our discussion:

“Irradiation is often considered a method to improve food safety without affecting organoleptic properties. However, this must be evaluated for each food product, and the irradiation dose and temperature during irradiation must be considered. Warming during irradiation has been associated with a reduction in organoleptic properties^34^. Increasing irradiation dose is also associated with a reduction in organoleptic properties^35^. Accordingly, lower doses of radiation are often preferred (< 5 kGy), which have been shown to continue to inactivate between 90 and 95% of pathogens^36^, thus maintaining food safety. **While the organoleptic properties of irradiated finfish were not specifically evaluated in this study, we did observe undesirable changes in color and odor.** For example, when our chilled salmon samples were irradiated at doses of 2 kGy, the pink color of the salmon meat turned dull, and the fish emitted a smell akin to cooked fish. Thus, like most previous work, irradiation dose and temperature should be carefully selected to maintain the organoleptic properties of finfish while ensuring a reduction in the density of pathogenic bacteria.”

In response to the reviewers' comment, we have removed this entire paragraph.

9. Line 213-227 Will the treatment cause adverse effects and within what range will it not cause adverse effects with references.

It is unclear what the reviewer is requesting. We think that they might be asking about organoleptic properties. Accordingly, we have added information about previous studies that have irradiated finfish within the doses we used and examined organoleptic properties post-irradiation. This can be found in the second last paragraph of the discussion.

**In conclusion, the reviewer made multiple fundamental errors in assessing our manuscript. The majority of the comments are outside our paper's scope, do not fundamentally affect the analysis, conclusions, or clarity of our manuscript, are nearly impossible to interpret, and are essentially comments asking for more work for the sake of asking for more work. Simply doing more experiments that do not impact the fundamental technical rigor, significance, or conclusions of a manuscript owing to the whims of a reviewer is not a review criterion established by PLoS One.**

Literature Cited

1	Bikle, D. D. Vitamin D: production, metabolism and mechanisms of action. (2015).

2	Zhang, Q.-F., Xiao, H.-M., An, N., Zhu, Q.-F. & Feng, Y.-Q. Determination of vitamin D metabolites in various biological samples through an improved chemical derivatization assisted liquid chromatography-tandem mass spectrometry approach. *Analytical Methods* **15**, 6009-6014 (2023).

3	Phillips, K. M., Pehrsson, P. R. & Patterson, K. Y. Survey of vitamin D and 25-hydroxyvitamin D in traditional native Alaskan meats, fish, and oils. *Journal of Food Composition and Analysis* **74**, 114-128 (2018).

4	Lu, Z. *et al.* An evaluation of the vitamin D3 content in fish: Is the vitamin D content adequate to satisfy the dietary requirement for vitamin D? *The Journal of steroid biochemistry and molecular biology* **103**, 642-644 (2007).

5	Teichert, M. & Horubala, A. The effects of gamma 60Co on vitamin D3 and certain sterols in foods. *Acta Alimentaria Polonica* **III** (1977).

6	van den Ouweland, J. M., Vogeser, M. & Bächer, S. Vitamin D and metabolites measurement by tandem mass spectrometry. *Rev Endocr Metab Disord* **14**, 159-184, doi:10.1007/s11154-013-9241-0 (2013).

7	Yeung, B. & Vouros, P. The role of mass spectrometry in vitamin D research. *Mass Spectrometry Reviews* **14**, 179-194 (1995).

8	Zelzer, S., Goessler, W. & Herrmann, M. Measurement of vitamin D metabolites by mass spectrometry, an analytical challenge. *Journal of Laboratory and Precision Medicine* **3** (2018).

9	El-Khoury, J. M., Reineks, E. Z. & Wang, S. Progress of liquid chromatography-mass spectrometry in measurement of vitamin D metabolites and analogues. *Clinical biochemistry* **44**, 66-76 (2011).

10	Higashi, T., Shimada, K. & Toyo’oka, T. Advances in determination of vitamin D related compounds in biological samples using liquid chromatography–mass spectrometry: A review. *Journal of Chromatography B* **878**, 1654-1661 (2010).

11	Gathungu, R. M., Flarakos, C. C., Satyanarayana Reddy, G. & Vouros, P. The role of mass spectrometry in the analysis of vitamin D compounds. *Mass Spectrometry Reviews* **32**, 72-86 (2013).

12	Volmer, D. A., Mendes, L. R. & Stokes, C. S. Analysis of vitamin D metabolic markers by mass spectrometry: current techniques, limitations of the “gold standard” method, and anticipated future directions. *Mass Spectrometry Reviews* **34**, 2-23 (2015).

13	Al-Kahtani, H. A. *et al.* Amino acid and protein changes in tilapia and Spanish mackerel after irradiation and storage. *Radiation Physics and Chemistry* **51**, 107-114 (1998).

14	Liu, M. S., Chen, R. Y., Tsai, M. J. & Yang, J. S. Effect of gamma irradiation on the keeping quality and nutrients of tilapia (Oreochromis mossambicus) and silver carp (Hypophthalmichthys molitrix) stored at 1. C. *Journal of the Science of Food and Agriculture* **57**, 555-563 (1991).

15	Underdal, B., Nordal, J., Lunde, G. & Eggum, B. effect of ionizing radiation on the nutritional value of mackerel. *LWT Lebensmitt Wissensch Technol* (1976).

16	Venugopal, V. Quality of proteins in raduarized Indian mackerel (Rastrelliger kanagurta): physicochemical evaluation. *Journal of Food Biochemistry* **5**, 145-154 (1981).

17	Özden, Ö. & Erkan, N. Impacts of gamma radiation on nutritional components of minimal processed cultured sea bass (Dicentrarchus labrax). *Iranian Journal of Fisheries Sciences* **9**, 265-278 (2010).

18	Brooke, R., Ravesi, E., Gadbois, D. & Steinberg, M. Preservation of fresh unfrozen fishery products by low-level radiation. 5. The effects of radiation pasteurization on amino acids and vitamins in haddock fillets. . (Bureau of Commercial Fisheries Tech. Lab., Glocester, Mass., 1966).

19	O'Neil, C. E., Keast, D. R., Fulgoni, V. L. & Nicklas, T. A. Food sources of energy and nutrients among adults in the US: NHANES 2003–2006. *Nutrients* **4**, 2097-2120 (2012).

20	Adam, S., Paul, G. & Ehlermann, D. Influence of ionizing radiation on the fatty acid composition of herring fillets. *Radiation Physics and Chemistry (1977)* **20**, 289-295 (1982).

21	Hammer, C. T. & Wills, E. The effect of ionizing radiation on the fatty acid composition of natural fats and on lipid peroxide formation. *International Journal of Radiation Biology and Related Studies in Physics, Chemistry and Medicine* **35**, 323-332 (1979).

22	Mbarki, R., Sadok, S. & Barkallah, I. Quality changes of the Mediterranean horse mackerel (Trachurus mediterraneus) during chilled storage: The effect of low-dose gamma irradiation. *Radiation Physics and Chemistry* **78**, 288-292 (2009).

23	Lakshmanan, R., Venugopal, V., Rao, B. & Bongirwar, D. Stability of lipids of Indian mackerel to gamma irradiation. *Journal of Food Lipids* **6**, 277-285 (1999).

24	Armstrong, S. G., Wyllie, S. G. & Leach, D. N. Effects of preservation by gamma-irradiation on the nutritional quality of Australian fish. *Food Chemistry* **50**, 351-357 (1994).

25	Thayer, D. Food irradiation: benefits and concerns. *Journal of food quality* **13**, 147-169 (1990).

26	Underdal, B., Nordal, J., Lunde, G. & Eggum, B. effect ionizing radiation on the nutritional value of fish (cod) protein. *Lebensmittel-Wissenschaft+ Technologie. Food science+ technology* (1973).

27	Subar, A. F., Krebs-Smith, S. M., Cook, A. & Kahle, L. L. Dietary sources of nutrients among US children, 1989–1991. *Pediatrics* **102**, 913-923 (1998).

28	Hau, L.-B. & Liew, M.-S. Effects of γ-irradiation and cooking on vitamins B6 and B12 in grass prawns (Penaeus monodon). *Radiation Physics and Chemistry* **42**, 297-300 (1993).

29	Aly, A., Emam, O. & Mohamed, E. Effects of gamma irradiation on salted and frozen mullet fish during storage period. *Food science and Quality Management* **23**, 1-9 (2014).

30	Javan, S. & Motallebi, A. A. in *Biological Forum.* 165 (Research Trend).

31	Kardashev, A. in *Food Irradiation. Proceedings of the International Symposium on Food Irradiation.*

32	Knapp, F. & Tappel, A. Comparison of the radiosensitivities of the fat-soluble vitamins by gamma irradiation. *Journal of agricultural and food chemistry* **9**, 430-433 (1961).

33	Prakash, S., Kailasam, S. & Patterson, J. Effect of radiation on the nutritional and microbial qualities of salted and sun-dried Sharpfin barracuda (Sphyraena acutipinnis). *International journal of radiation biology* **91**, 540-546 (2015).

34	Diehl, J. F. *Safety of irradiated foods*. 2nd Edition edn, 43-88 (CRC Press, 1995).

35	Ferdousi, R. *et al.* Effects of Gamma Irradiation on Physicochemical and Sensory Properties of Cooked Beef Sausages. *Philippine Journal of Science* **151** (2022).

36	Ashie, I. N. A., Smith, J. P., Simpson, B. K. & Haard, N. F. Spoilage and shelf‐life extension of fresh fish and shellfish. *Critical Reviews in Food Science and Nutrition* **36**, 87-121, doi:10.1080/10408399609527720 (1996).
