# Supplementary material for: The effect of gamma irradiation on the stability of vitamin D in select finfish species
Source: PLoS One. 2025 Sep 26;20(9):e0332867. doi: 10.1371/journal.pone.0332867 (PMC12469152; doi:10.1371/journal.pone.0332867)
Supplement: Attachment — Submitted filename: Response to Reviewers.docx [file pone.0332867.s003.docx]

We sincerely thank the reviewers for their thoughtful and constructive feedback on our manuscript. We have carefully considered each comment and have revised the manuscript accordingly. Below, we have copied each reviewer comment in full and provided our detailed response directly beneath each one. Changes made to the manuscript are indicated where applicable, and line numbers have been provided to identify the location of each change. Importantly, the line numbers reflect lines in the marked up version of the manuscript, and not the clean version of the manuscript.

Reviewer #2:

**Abstract**

Q.1 What was the duration of the study or trial period during which samples were monitored or stored?

The research was performed over a nine-month period.

- Irradiation: Late October 2022
- Vitamin D extraction
  - Salmon, chilled: Late November to late December 2022
  - Salmon, frozen: Late October to late November 2022
  - Trout, frozen: January 2023
  - Trout, chilled: February to mid-March 2023
- LC-MS analysis
  - Salmon, chilled: Late January 2023
  - Salmon, frozen: Late January 2023
  - Trout, frozen: Late January 2023
  - Trout, chilled: Late June 2023

However, we feel as though this timeline is not relevant to the findings or conclusions of our manuscript. Thus, we have not modified the abstract.

Q.2 How many samples were used per species and per treatment condition (irradiation dose, temperature)?

Three filets (or samples) were used for each species and temperature combination (e.g., three filets of chilled salmon), totaling 12 filets for the four combinations. Each filet was dissected into 4 portions, and each portion was irradiated with a different dose of gamma irradiation. We added a clarifying sentence to the abstract (Lines 132-134).

**Introduction**

Q.3 Furthermore, irradiating chicken, beef, lettuce, and crustaceans is already commonplace in the United States. Add references (Line 49-50).

A reference from the FDAs approval of gamma irradiation was added to the manuscript (Line 179).

Q.4 In 2021, Americans consumed an average of 20.5 pounds of finfish and shellfish, representing the highest average consumption weight reported to date. How much of this consumption is specifically attributable to finfish alone? Is it appropriate to generalize this statistic to support an argument focused only on finfish? (Line 27)

We acknowledge that the reported figure of 20.5 pounds includes both finfish and shellfish; however, we chose to include this statistic because it is a widely reported and an authoritative measure of U.S. seafood consumption trends. While it does not distinguish between finfish and shellfish, it effectively illustrates the broader point that seafood consumption is increasing in the United States.

Q.5 Irradiation causes DNA damage to kill bacteria, but how does this vary across different bacterial genera mentioned (e.g., Vibrio vs. Clostridium)? Are there species-specific resistance thresholds to irradiation? (Line 45-46)

The references included in the manuscript were intended to demonstrate that the use of gamma irradiation to eliminate bacterial pathogens is a well-established and accepted practice. While we recognize that bacterial resistance to irradiation can vary by species and genus, a detailed examination of species-specific resistance thresholds was beyond the scope of our study.

Nevertheless, our primary objective was to evaluate whether gamma irradiation adversely affects the nutritional components of fish, rather than to assess its differential efficacy across bacterial genera. A sentence was added to the introduction to specify this objective (Lines 180-183)

Q.6 What is the rationale for testing both chilled and frozen conditions?

Commercial fish products are sold either fresh (chilled) or frozen and we sought to test the stability of vitamin D for both types of products. A sentence was added to the introduction to explain our choice for irradiating at chilled and frozen conditions (Lines 190-195).

Additionally, it has also been well-established that reducing the temperature of irradiation can reduce the effects of irradiation on changes in nutrition and microbial load. Thus, we chose to examine the impacts of irradiation at these two temperatures. Two sentences were added to the introduction that mentions this occurrence and why we chose two ranges of doses for chilled and frozen samples (Lines 173-175).

**Methodology**

Q.7 How many individual fish were used per species for the entire experiment? The method only mentions "three dissected fish. (Line 75)

Three filets (or samples) were used for each species and temperature combination (e.g., three filets of chilled salmon), totaling 12 filets for the four combinations. Each filet was dissected into 4 portions, and each portion was irradiated with a different dose of gamma irradiation.

Q.8 Were all pieces taken from the same fish used as replicates for each dose, or were they from different fish? If from the same fish, how do the authors justify the use of pseudo-replication in the statistical analysis?

To account for biological variability among fish samples and to standardize comparisons across irradiation doses, we selected three filets from three different individual fish and dissected each into four portions. Each piece was then subjected to a different irradiation dose. This approach allowed us to monitor relative changes in vitamin D concentration within the same filet, using the non-irradiated portion as the control (set at 100%). By doing so, we minimized inter-sample variability and ensured that observed differences were attributable to irradiation rather than natural variation among individual fish. This has been clarified in the Data Analysis subsection of the Materials and Methods section (Lines 288-291).

Q.9 Why were different dose ranges used for chilled (0–2 kGy) and frozen (0–4 kGy) conditions? Is there literature or preliminary data justifying this discrepancy? Add reference (Line 83)

Previous studies show that gamma irradiation is less effective at killing bacteria at lower temperatures, requiring higher doses at subfreezing conditions [1]. These ranges are also consistent with previous work that has examined the impacts of irradiation on finfish, as well as irradiation doses currently used to treat finfish in international markets. Two sentences were added to the introduction that mentions this occurrence and why we chose two ranges of doses for chilled and frozen samples (Lines 173-175).

Q.10 How were the chilled (0°C) and frozen (-17°C) conditions monitored and maintained during irradiation? (Line 80)

All irradiation was performed at Gateway America, which routinely irradiates food for commercial distribution. During the irradiation process, probes that monitor temperature and dose are inserted amongst the irradiated samples. All irradiation occurs in a large, refrigerated warehouse, which assists to ensure that fluctuations in temperature do not occur. We have clarified this in the Methods section (Lines 226-227)

Q.11 What was the rationale for using vitamin D3-d3 as the internal standard at 50 µL (2 ng/µL)? Was the spike level optimized to fall within the linear range of the LC-MS/MS?

During initial method development, a larger volume of internal standard (IS) was added to each sample. However, we observed that this produced a disproportionately large IS signal relative to the vitamin D3 signal in fish extracts, which could interfere with accurate quantitation. As a result, we optimized the IS spike volume to 50 µL at 2 ng/µL to ensure that the IS concentration was comparable to the analyte concentration in typical samples. This adjustment provided a more balanced signal, minimized the risk of ion suppression or detector saturation, and ensured accurate, reproducible quantitation within the linear dynamic range of the method.

Q.12 Samples were stored at –4°C after irradiation. This is an unusually high "freezer" temperature for sample preservation. Could vitamin D degradation have occurred prior to extraction and analysis? (Line 88)

Previous studies have shown that vitamin D3 is more stable at lower temperatures, with significantly greater stability reported at 4 °C compared to 27 °C [2]. Thus, we are confident that storing our samples at –4 °C, a subfreezing temperature, did not lead to degradation of vitamin D3 prior to extraction and analysis. Although –4 °C is slightly higher than typical ultra-low freezer settings (e.g., –20 °C), it is still below the threshold at which vitamin D degradation has been reported under standard storage conditions.

Q.13 Was the assumption of homoscedasticity (equal variance) checked before applying ANOVA? If heterogeneity of variance was present, why was ANOVA used rather than a non-parametric test or transformation?

As noted in our original submission the distribution of all vitamin D for each finfish species and irradiation temperature was assessed using a Shapiro-Wilk test for normality. Thus, an ANOVA followed by a Tukey HSD is highly appropriate. We have updated our manuscript to include P values for each finfish species and temperature for the Shapiro-Wilk test in the Methods section (Lines 293-296).

**Results and Discussion**

Q.14 The data are reported as mean ± SEM. How many technical replicates per biological replicate were run to ensure analytical precision?

To ensure analytical precision, randomly selected samples were run in duplicate as technical replicates. This approach allowed us to confirm consistency in measurement across runs without significantly depleting the limited sample volume available for analysis. However, because technical replicates were not consistently run for each sample, we only used biological replicates for our analysis.

Q.15 Why does 1 kGy in chilled salmon show a greater loss (60%) than 2 kGy (53%)? (Line 144)

While it would appear that 1 kGy showed a great loss as compared to 2 kGy, these data points are in fact not different from each other. A two-tailed t-test comparing vitamin D isolated from chilled salmon irradiated at 1kGy and 2kGy did not show significance (P = 0.44). The averages are also well within the error range as noted by the overlapping error bars.

Q.16 Author hypothesized a matrix effect protects vitamin D in trout but not salmon. But no lipidomic, proteomic, or moisture content comparisons made between the two species to support this claim.

Performing lipidomic, proteomic or moisture content analysis is beyond the scope of this study. However, according to the USDA FoodData Central [3], farmed salmon and trout have similar protein content (20.4 g and 19.9 g per 100 g, respectively), but differ notably in fat and water content. Salmon contains more than twice the fat (13.4 g vs. 6.18 g) and less water (64.6 g vs. 73.8 g) than trout. While we did not directly measure these properties in our samples, we proposed that such compositional differences—along with the presence of skin on one side of the trout fillets versus fully skinned salmon fillets—may help explain the species-dependent differences observed in vitamin D stability following irradiation. These details have been added to the Results and Discussion section (Lines 417-422).

Q.17 2 kGy dose in frozen salmon retained more vitamin D than the chilled equivalent, what are the implications of partial thawing or ice crystal formation during irradiation?

We did not observe thawing or ice crystal formation during irradiation, as the temperature was kept constant. It is expected that Vitamin D stability is improved at lower temperatures, which has been previously demonstrated for other compounds during irradiation.

Q.18 Authors should perform Two-way ANOVA to compare the effects of species and temperature simultaneously? A full factorial analysis could better reveal interaction effects.

Unfortunately, we cannot run a two-way ANOVA across our entire data set as only 0 kGy and 2kGy were the only consistently tested irradiation doses tested between both temperatures.

We did, however, perform a two-way ANOVA using the 2 kGy data. Because a Shapiro-Wilk test performed on only the 2kGy indicated that it was not normally distributed (P < 0.0001), we log-transformed the data to achieve normality. We then performed a two way ANOVA considering both species and temperature. Our findings indicate that there is a significant effect of both finfish species and temperature on the stability of vitamin D (P = 0.006). This analysis has been added to the Materials and Methods section (Lines 297-315) and the results included in the Results and Discussion section (Lines 384-386).

Q.19 Author mentioned that previous work showing no vitamin D loss at 5 kGy in sharpfin barracuda. Could this species-specific protection due to matrix effects, or are methodological differences?

It could be either.

The methods used in the article noted above were not well described and simply stated that an 1990 version of the AOAC official method was used. Thus, it is no clear exactly clear what they performed and how it might differ from our extraction and analysis methods. Additionally, the barracuda was sun-dried and had a moisture content of 24.6%, while our samples were raw and had water content of 64.6% for salmon and 73.8% for trout [3].

We also note that it could be a species specific difference due to matrix effects. For example, non-irradiated sharpfin barracuda has 7.09% lipids, 7.09g/100g or lipids. This contrasts to 13.4 g vs. 6.18 g of fat per 100g observed in salmon and trout, respectively [3].

Q.20 Author must assess the microbial load post-irradiation to confirm pathogen reduction. If not, how this manuscript confidently mentioned that irradiation at appropriate intensities can reduce the pathogenic burden on finfish while maintaining the concentration of vitamin D? (Line 230).

It was not the goal of this work to demonstrate the microbial reduction potential of irradiation, as it has been demonstrated extensively in other studies [4-11]. This literature supports that doses much lower than the doses used in this study effectively reduce or eliminate microbial load. This study was solely focused on the effects of irradiation on Vitamin D.

We have modified to statement in the results/discussion section to make it clear that our claim is based on previous literature, and not examined in this manuscript. We have also noted that this should be examine in a future study (Lines 451-456)

**References**

1.	Radomyski T, Murano EA, Olson DG, Murano PS. Elimination of pathogens of significance in food by low-dose irradiation: a review. Journal of food protection. 1994;57(1):73–86.

2.	Zareie M, Abbasi A, Faghih S. Influence of storage conditions on the stability of vitamin D3 and kinetic study of the vitamin degradation in fortified canola oil during the storage. Journal of Food Quality. 2021;2021(1):5599140.

3.	United States Department of Agriculture ARS. FoodData Central 2019 [cited 2025]. Available from: fdc.nal.usda.gov.

4.	Dion P, Charbonneau R, Thibault C. Effect of ionizing dose rate on the radioresistance of some food pathogenic bacteria. Canadian journal of microbiology. 1994;40(5):369–74.

5.	Ahmed IO, Alur MD, Kamat AS, Bandekar JR, Thomas P. Influence of processing on the extension of shelf‐life of Nagli‐fish (Sillago sihama) by gamma radiation. International journal of food science & technology. 1997;32(4):325–32.

6.	Jakabi M, Gelli DS, Torre JC, Rodas MA, Franco BD, Destro MT, et al. Inactivation by ionizing radiation of Salmonella enteritidis, Salmonella infantis, and Vibrio parahaemolyticus in oysters (Crassostrea brasiliana). Journal of food protection. 2003;66(6):1025–9.

7.	Matches J, Liston J. Radiation destruction of Vibrio parahaemolyticus. Journal of Food Science. 1971;36(2):339–40.

8.	Ama AA, Hamdy M, Toledo R. Effect of heating, pH and thermoradiation on inactivation of Vibrio vulnificus. Food Microbiology. 1994;11(3):215–27.

9.	Torres Z, Kahn G, Vivanco M, Guzman G, Bernuy B. Shelf-life extension and decontamination of fish fillets (Trachurus picturatus murphyi and Mugil cephalus) and shrimp tails (Penaeus vannamei) inoculated with toxigenic Vibrio cholerae O1 El Tor using gamma radiation. Irradiation to control Vibrio infection from consumption of raw seafood and fresh produce IAEA-TECDOC. 2001;1213:47–56.

10.	Kamat A, Thomas P. Radiation inactivation of some food‐borne pathogens in fish as influenced by fat levels. Journal of applied microbiology. 1998;84(4):478–84.

11.	Rajkowski KT. Radiation D 10-values on thawed and frozen catfish and tilapia for finfish isolates of Listeria monocytogenes. Journal of food protection. 2008;71(11):2278–82.
