# Supplementary material for: The effect of gamma irradiation on the stability of vitamin D in select finfish species
Source: PLoS One. 2025 Sep 26;20(9):e0332867. doi: 10.1371/journal.pone.0332867 (PMC12469152; doi:10.1371/journal.pone.0332867)
Supplement: Attachment — Submitted filename: Response_to_Reviewers_auresp_3.docx [file pone.0332867.s004.docx]

**Response to Reviewers**

Reviewer/Editor Comment 1

“I recommend a final check to ensure that all references cited in the text are properly included in the Literature Cited section, and that the formatting is consistent throughout.”

Response:

We thank the reviewer for this important suggestion. We undertook a comprehensive review of the reference list and performed the following steps:

1. Cross-check of in-text citations: Every reference cited in the manuscript text was verified against the References section. All in-text citations are now included in the reference list, and no extraneous references remain.
2. Consistency in formatting: References have been reformatted to comply fully with PLOS ONE requirements (Vancouver style):

- All journal titles are now given in full title case (e.g., Journal of Food Protection rather than J Food Prot.).
- Author initials have been standardized without periods (e.g., Smith AB rather than Smith A.B.).
- Page ranges have been expanded (e.g., S391–S395 rather than S391–S5).
- Organizational reports and government documents (e.g., WHO/FAO, USDA, NOAA, CFR) have been reformatted to include full organizational names, place of publication, publisher, year, URL, and access date.
- Book chapters and monographs (e.g., Thayer DW et al. 1991; Diehl JF. 1995) have been revised to include editors, edition, publisher, location, and page ranges.
- Electronic resources (e.g., USDA FoodData Central) now include full URLs and access dates in the proper format ([cited 2025 Apr 18]).

3. Verification of accuracy: We cross-checked all DOIs, PMIDs, and page numbers to ensure accuracy. We also confirmed that no cited articles had been retracted.

These corrections have been applied consistently across all 46 references. We believe this addresses the reviewer’s concern fully.
